# Peptide with Dual Roles in Immune and Metabolic Regulation: Liver-Expressed Antimicrobial Peptide-2 (LEAP-2)

**DOI:** 10.3390/molecules30020429

**Published:** 2025-01-20

**Authors:** Yitong Li, Ying Liu, Meng Gou

**Affiliations:** 1 College of Life Science, Liaoning Normal University, Dalian 116081, China; jiantangwysa1@163.com; 2 Lamprey Research Center, Liaoning Normal University, Dalian 116081, China; 3 Haixia Institute of Science and Technology, College of Horticulture, Fujian Agriculture and Forestry University, Fuzhou 350007, China; ying.liu@fafu.edu.cn

**Keywords:** LEAP-2, antimicrobial activity, GHSR1a, ghrelin, metabolic regulation, clinical potential

## Abstract

Liver-expressed antimicrobial peptide 2 (LEAP-2) was originally discovered as an antimicrobial peptide that plays a vital role in the host innate immune system of various vertebrates. Recent research discovered LEAP-2 as an endogenous antagonist and inverse agonist of the GHSR1a receptor. By acting as a competitive antagonist to ghrelin, LEAP-2 influences energy balance and metabolic processes via the ghrelin–GHSR1a signaling pathway. LEAP-2 alone or the LEAP-2/ghrelin molar ratio showed potential as therapeutic targets for obesity, diabetes, and metabolic disorders. This review explores the recent advances of LEAP-2 in immune modulation and energy regulation, highlighting its potential in treating the above diseases.

## 1. Introduction

Antimicrobial peptides (AMPs) are naturally occurring small molecules found in various organisms. These peptides exhibit broad-spectrum antibacterial and antifungal activity and play a key modulatory role in innate immunity [1,2]. Two liver-expressed antimicrobial peptides (LEAP-1 and LEAP-2) have been isolated from human blood. They are characterized by either four or two pairs of disulfide bonds, respectively, and encoded by three exons and two introns. LEAP-1, the first blood-derived antimicrobial peptide to be extensively studied, is involved in both innate immune defense and the regulation of iron homeostasis, earning it the additional name hepcidin [3,4].

LEAP-2, first isolated from human blood ultrafiltrate in 2003, was identified as a novel mammalian cysteine-rich and cationic secretory peptide, marking it as the second blood-derived antimicrobial peptide [5]. Compared to LEAP-1, research on LEAP-2 has been relatively limited. LEAP-2 plays a critical role in the innate immune system by defending against pathogenic invasion. Its effective antibacterial concentrations have been measured in the range of hundreds of μM, much higher than physiological levels [6,7]. In addition, LEAP-2 has been shown to modulate fibroblast growth factor signaling in the amphibian *Xenopus laevis*, marking the first report on the involvement of LEAP-2 in animal cell signaling [8].

Additionally, LEAP-2 has been identified as an endogenous antagonist and inverse agonist of growth hormone secretory receptor 1a (GHSR1a). While ghrelin, with acyl modification, is the primary endogenous ligand for GHSR1a, LEAP-2 modulates the ghrelin–GHSR1a signaling pathway, influencing energy metabolism, including growth hormone secretion, food intake, and the regulation of glucose homeostasis [9,10]. Although originally discovered as an antimicrobial peptide, the function of LEAP-2 as a GHS-R1a has only recently been recognized. LEAP-2 has been identified across a wide range of species, including birds, reptiles, amphibians, and fish. Its effects are species-dependent and influenced by its structure, including protection against pathogenic bacteria, the inhibition of food intake, control of blood glucose levels and weight loss, and more [11,12,13,14,15].

However, a systematic classification and summary of LEAP-2 research progress is still lacking. In this review, we summarize the evolutionary diversity and functional characteristics of LEAP-2 across species, analyze its therapeutic potential in treating obesity, diabetes, addiction, and other diseases, and explore its potential clinical applications and limitations.

## 2. LEAP-2 Evolutionary History

### 2.1. Mammals

Since its initial purification from human blood, LEAP-2 has been reported to be highly conserved among mammals, including monkeys, mice, cattle, pigs, and even domestic dogs. While primarily expressed in the liver, LEAP-2 is also produced in other tissues and organs [5]. Human LEAP-2 is initially synthesized as a 77-amino-acid-residue precursor, which is then processed into a mature 40-amino-acid-residue peptide [6]. The mouse LEAP-2 cDNA encodes a 76-amino-acid protein that shares 83% sequence similarity with the human LEAP-2 precursor, while the mature peptide sequence is identical between the two species, consisting of 40 amino acid residues [5]. In pigs, the LEAP-2 cDNA sequence contains 525 base pairs, encoding a predicted peptide of 77 amino acid residues. The porcine LEAP-2 precursor shares over 80% sequence homology with its human, bovine, mouse, and rhesus monkey counterparts, with the mature peptides sharing 95–97% sequence similarity [16].

### 2.2. Birds, Amphibians, and Reptiles

In addition to mammals, the first non-mammalian form of LEAP-2 was described in chickens. The chicken LEAP-2 precursor encodes a peptide of 76 amino acid residues that is 59% identical to human LEAP-2, though only 16.6% pro-peptide amino acids are identical to the human counterpart [17,18]. Reptiles, the oldest amniotes, possess an ancient innate immune system with both identified and predicted antimicrobial peptides. Although the predicted LEAP-2 gene of many reptiles has been annotated, the antimicrobial activity or other physiological functions of this peptide remain largely unstudied [14]. In amphibians, both *Xenopus tropicalis* and *Xenopus laevis* express LEAP-2 proteins comprising 80 amino acid residues, which share only 50% homology with human LEAP-2. However, the mature peptides of 40 amino acid residues are well-conserved, displaying 67.5% homology to the human LEAP-2 sequence [8].

### 2.3. Fishes

**Figure 1:**
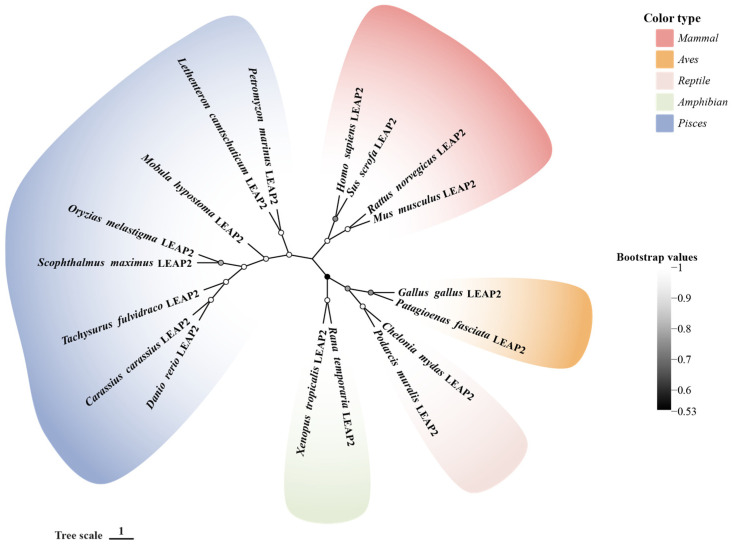
Phylogenetic relationship of the LEAP-2 gene in vertebrate. The phylogenetic tree of 18 full-length protein sequences was constructed by MEGA 11. The phylogenetic tree was established by the neighbor-joining method, bootstrapping with a parameter of 1000 repeats, the pairwise deletion method, and the p-distance selection method. GenBank accession numbers of the selected sequences are listed in Table 1.

LEAP-2 orthologs have also been identified from a variety of fish species, especially teleost fish, which represent the most diverse vertebrate group and have been extensively studied in comparative immunology [19,20]. Unlike mammals, which have only one LEAP-2, teleost fish have three LEAP-2 paralogs, namely LEAP-2A, LEAP-2B, and LEAP-2C, each exhibiting distinct tissue expression patterns [20,21,22]. In most teleost fish, both LEAP-2A and LEAP-2B mature peptides contain 41 amino acid residues. A phylogenetic analysis indicated that teleost LEAP-2 clusters distinctly from other species, consistent with evolutionary divergence, and further separates into two branches, with species like trout and carp forming one and *Acanthopterygii* forming the other [23]. In *Chondrichthyes*, LEAP-2B and LEAP-2C sequences have been identified and characterized, showing homology to LEAP-2s from other fish species [22]. Additionally, LEAP-2 has been identified in the jawless vertebrate lamprey, a key species for studying immune system evolution, which encodes an 87-amino-acid peptide. Although homology with higher vertebrates is low, the gene structure of LEAP-2 in lampreys is relatively complete and highly conserved, of which the mature peptide has four cysteine residues, capable of forming a core structure of two disulfide bonds. It has been found that lamprey LEAP-2 has a wide range of antimicrobial properties and dose-dependent antimicrobial mechanisms, but its antibacterial efficiency is relatively low compared with other vertebrates, and no homologues of LEAP-2 have been found in lower chordates, suggesting that LEAP-2 may have originated in ancient fish [24,25]. The cleavage site (RXXR) between the pro-peptide and the mature peptide of LEAP-2 is conserved in evolution from fish to mammals [20]. The phylogenetic relationship of vertebrate LEAP-2 is shown in Figure 1. All the sequences used for the phylogenetic analysis are listed in Table 1.

## 3. The Antimicrobial and Immunoregulatory Activities of LEAP-2

LEAP-2 was originally reported to have antimicrobial activity and proposed to be a critical component of the vertebrate innate immunity system, showing activity against a broad range of pathogens, including Gram-positive bacteria, Gram-negative bacteria, parasites, and fungi in vitro [26,27,28]. Its structural organization consists of three primary elements: a signal peptide, a pro-peptide, and a mature peptide. The sequences of mature peptides display more conservation across species than those of the signal peptides and pro-peptides. The central core of the LEAP-2 mature peptide is stabilized by two pairs of disulfide bonds consisting of four cysteine residues, which not only maintain its tertiary structure but also exert potent antimicrobial activity by creating pores that disrupt the cell envelope, leading to the leakage of cell contents and cell death [6,29]. However, linear LEAP-2 has been shown to be more efficient at penetrating cell membranes and binding to DNA than oxidized LEAP-2 with chemically synthesized disulfide bonds, which has been validated in both humans and fish [7,30].

LEAP-2 is predominantly expressed in the human liver, where it is secreted into the bloodstream and subsequently processed and eliminated by the kidney. It shows relatively high expression in the liver, with differential expression across other tissues such as the stomach, intestinal tissues, heart, and even the central nervous system (CNS) of mammals. A LEAP-2 metabolite was also identified in urine of humans and domestic dogs [5,31]. The LEAP-2 gene was identified to be highly expressed in human lung adenocarcinoma epithelial cells infected by Middle East respiratory syndrome coronavirus (MERS-CoV), suggesting its potential as a target for treating viral infections [32]. Dental caries and periodontitis, as inflammatory processes, are the top two common diseases that cause tooth loss in the world. They are related to plaque accumulation and dysbiosis of the oral microbiota and can also be caused by immune-related diseases, diabetes, and other factors. The latest treatment involves probiotics, which can produce antibacterial substances for further immune regulation [33,34,35,36]. The similarity between LEAP-2 and probiotics is also expected to provide novel insights into the treatment of oral diseases. In chickens, LEAP-2 is expressed in the epithelial tissues, particularly the liver, where it acts as a barrier to pathogenic microorganisms by preventing their penetration through the epithelial surfaces, forming a critical component of the innate immune defense system [18]. Amphibian LEAP-2 disrupts bacterial cell membranes and hydrolyzes bacterial gDNA, consistent with mammalian studies; however, the highest expression was found in the kidney, followed by the liver [11].

More research on the antimicrobial activity of LEAP-2 has been focusing on fish. It can enhance the immune response by preventing an overreacting inflammatory response through chemotaxing monocytes/macrophages, increasing the respiratory burst of these immune cells, and inhibiting the expression of inflammatory factors (e.g., TNF-α and IL-1β). LEAP-2 also can regulate other immune signaling pathways during infection, such as the Toll-like receptor (TLR) and NOD-like receptor signaling pathways [12,37]. In teleost and cartilaginous fish, LEAP-2 has been characterized by high expression in the liver and kidney, with expression levels often increasing significantly in response to bacterial infection [20,22]. However, LEAP-2 expression in lampreys differs significantly from other species, with minimal expression in the liver and prominent expression in muscle, the supraneural body, and the buccal gland, reflecting the unique immune adaptations in this ancient jawless vertebrate [25]. Recently, additional antimicrobial mechanisms, including the iron apoptosis signaling pathway, have been identified in fish. Loss of LEAP-2 may accelerate the iron death process by regulating the expression of associated genes, such as antioxidant enzymes and iron transporters. This mechanism may further exacerbate the inflammatory response and tissue damage during infection. Furthermore, despite the strong antimicrobial activity of LEAP-2 and its analogues of *Larimichthys crocea*, transcriptome data revealed downregulation of its expression during *Cryptocaryon irritans* infection. This suggests that LEAP-2 may be involved in negatively regulating or modulating other immune responses during infection, in contrast to other antimicrobial peptides [38]. These studies of mechanisms and signaling pathways present new therapeutic opportunities for the prevention or treatment of bacterial infections in aquaculture [12,20,39,40].

## 4. LEAP-2 Plays an Important Role in Energy Metabolism by Regulating the Ghrelin–GHSR1a Signaling System

In addition to its widely described function in innate immune response, LEAP-2 has been shown to act as an antagonist and inverse agonist of GHSR1a, a G-protein coupled receptor highly expressed in the hypothalamus and anterior pituitary [10,41,42]. Studies have shown that LEAP-2 can stabilize the conformation of GHSR1a and exert activity toward GHSR1a through the interaction of the N-terminal region with the enrichment of hydrophobic side-chains [43]. In contrast, ghrelin, a stomach-derived hormone, acts as the endogenous agonist of GHSR1a. In its acylated form, catalyzed by ghrelin-O-acyltransferase (GOAT), ghrelin is involved in maintaining body energy metabolism by regulating GHSR1a to promote appetite, maintaining blood glucose levels, and controlling body weight [9]. According to the latest research, LEAP-2 and ghrelin can bind to GHSR1a in both a competitive and non-competitive manner. LEAP-2 behaves as a competitive antagonist when added simultaneously with ghrelin and as a non-competitive antagonist when added before ghrelin, possibly due to its slow dissociation from the receptor GHSR1a [10,42].

**Figure 2:**
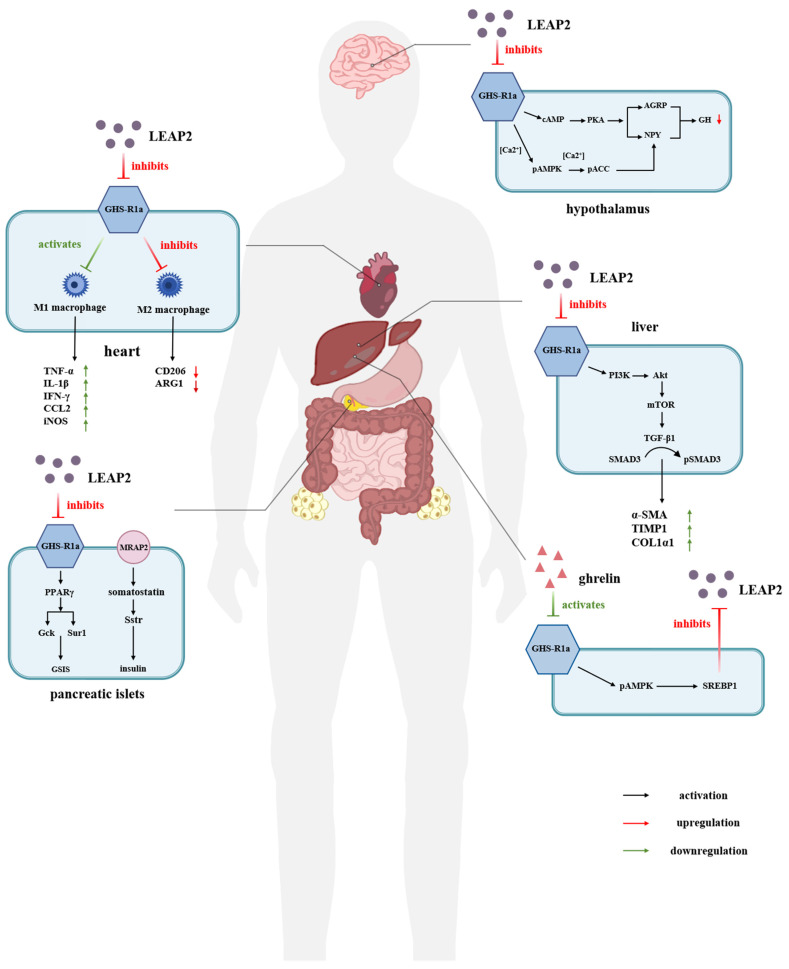
Schematic representation of metabolic or disease regulation by LEAP-2 antagonism of GHSR1a in human organs. Mechanism of antagonism between ghrelin and LEAP-2: ghrelin inhibits the expression of SREBP1 through AMPK phosphorylation, thus decreasing LEAP-2 expression. In the hypothalamus, LEAP-2 inhibits the cAMP-PKA pathway on NPY/AgRP neurons and phosphorylates AMPK and ACC on NPY neurons, respectively, which reduces food intake and suppresses appetite, resulting in decreased GH secretion. In the heart, LEAP-2 upregulates the levels of pro-inflammatory cytokines as well as M1 macrophage markers by promoting M1 macrophage polarization, while inhibiting M2 macrophage polarization and downregulating its markers, and knockdown of LEAP-2 contributes to the treatment of obesity-induced myocardial injury. In the liver, hepatic fibrosis due to bariatric surgery is upregulated by LEAP-2 through the PI3K/AKT/mTOR signaling pathway, ultimately leading to the expression of pro-fibrotic factors. In pancreatic islets, LEAP-2 enhances GSIS by upregulating insulin-secretion-related genes through the GHSR1a-PPARγ signaling pathway. Simultaneously, LEAP-2 reverses the insulin inhibitory effect caused by ghrelin via GHSR1a and Mrap2 receptors. LEAP-2, liver-expressed antimicrobial peptide-2; GHS-R1a, growth hormone secretagogue receptor 1a; cAMP, cyclic adenosine monophosphate; PKA, protein kinase A; AGRP, Agouti-related protein; NPY, neuropeptide Y; AMPK, Adenosine 5′-monophosphate (AMP)-activated protein kinase; ACC, Acetyl CoA carboxylase; TNF-α, tumor necrosis factor-α; IL-1β, interleukin-1β; IFN-γ, Interferon-γ; CCL2, chemokine (C-C motif) ligand 2; iNOS, inducible nitric oxide sythase; CD206, Mannose receptor; ARG1, Arginase 1; PPARγ, peroxisome proliferator-activated receptor; Gck, glucokinase; Sur1, Sulfonylurea receptor; MRAP2, Melanocortin 2 Receptor Accessory Protein 2; Sstr, Somatostatin receptor; PI3K, Phosphoinositide 3-kinase; Akt, protein kinase B; mTOR, mammalian target of rapamycin; TGF-β1, transforming growth factor-β1; SMAD3, SMAD family member 3; α-SMA, Alpha Smooth Muscle Actin; TIMP1, AMP-activated protein kinase; COL1α1, Collagen type I α1; SREBP1, Sterol regulatory element-binding protein 1. Black arrows represent activation, green arrows represent upregulation and red arrows represent downregulation.

Therefore, LEAP-2 can prevent the binding of ghrelin by binding to GHSR1a and acts as a potent inhibitor of ghrelin in vivo [44,45,46]. This dual role allows LEAP-2 to play a pivotal part in regulating energy metabolism through the ghrelin–GHSR1a signaling pathway. Furthermore, LEAP-2 may offer therapeutic potential by modulating ghrelin-related responses under both normal and pathological conditions [43,47]. The regulation and effects of LEAP-2 antagonism against GHSR1a on human organ metabolism or disease are shown in Figure 2.

### 4.1. LEAP-2 in Relation to Food Intake and Appetite

Recent studies suggested that LEAP-2 and ghrelin exert opposing effects on food intake regulation, both of which are influenced by body weight and feeding status [48,49,50]. It has been shown that LEAP-2 hyperpolarizes and prevents acyl-ghrelin from activating arcuate NPY neurons, thereby confirming its antagonistic effect on ghrelin [51]. Thus, LEAP-2 can inhibit the central function of ghrelin via the liver–stomach–hypothalamic axis and modulate its action in response to changes in environmental conditions, further influencing food intake [51,52]. Recently, in vivo experiments have revealed that LEAP-2 functions is an effective anorexic agent, capable of blunting ghrelin-induced bulimia [53]. Hagemann et al. reported that in wild-type mice and GHSR-deficient mice, exogenous LEAP-2 was able to reduce growth hormone (GH) concentrations and reduce food intake [13]. In addition to the wild-type mice, Shankar et al. found that reduced LEAP-2 levels in high-fat-diet-fed female mice made them more sensitive to the effects of acyl-ghrelin on food intake and GH secretion. However, this effect was not observed in male mice, suggesting potential sex-specific differences that require further study [46].

Fasting or diet-induced weight loss can reverse the increase in plasma LEAP-2 levels in obese mice, indicating that the plasma LEAP-2/acyl-ghrelin molar ratio is a critical factor regulating the ghrelin–GHSR1a signaling pathway in response to changes in body weight, food intake, and appetite [51]. Since then, research on the interaction between diet and LEAP-2 has expanded, particularly with a focus on its role in the liver and intestines [54,55]. Bhargava et al. found that an increase in postprandial plasma LEAP-2 of humans without obesity was involved in the homeostasis of the body energy balance by promoting a decrease in postprandial appetite and food intake, as well as correlated negatively with body mass index (BMI), although no significant trends were observed for the plasma acyl-ghrelin or LEAP-2/acyl-ghrelin molar ratio [54]. LEAP-2 is predominantly expressed in the liver, and studies have examined its response to dietary changes. LEAP-2 mRNA levels in the liver of mice showed no significant increase after 3 weeks of high-fat-diet feeding. However, after 9 weeks of high-fat feeding, LEAP-2 expression increased significantly and returned to baseline levels after switching back to a standard diet. Additionally, a high-fat diet was found to induce resistance to LEAP-2 analogues, suggesting a modulation in LEAP-2 function under prolonged high-fat feeding conditions [56].

Interestingly, elevated LEAP-2 levels have been observed in patients with anorexia nervosa, which suggested an abnormal adaptation of the ghrelin–GHSR1a signaling pathway in these patients, though the underlying mechanism remains unresolved [57]. Ragland and Malin reported that a low-calorie diet reduced fasting LEAP-2 in women with obesity, regardless of exercise, while a low-calorie diet without exercise increased postprandial LEAP-2, contrary to the hypothesis that exercise reduces appetite. The trends in LEAP-2 and acyl-ghrelin levels were similar, though the exact proportion remained unclear, indicating that LEAP-2 may regulate appetite and metabolic parameters through mechanisms similar to ghrelin [58]. Meanwhile, Pedersen et al. reported that LEAP-2 concentrations decreased after prolonged fasting in humans. They found that the oral administration of glucose and lactate was more effective at increasing LEAP-2 than intravenous injection alone, with lactate, in particular, having a more significant effect, emphasizing the potential impact of clinical studies on appetite regulation [59].

### 4.2. LEAP-2 Regulation of Blood Glucose

In addition to its role in regulating appetite and body weight, ghrelin can increase plasma glucose levels by activating GHSR1a after food intake and modulating insulin secretion by islet cells [13,60]. Given the antagonistic relationship between ghrelin and LEAP-2, it is likely that LEAP-2 also plays a role in glucose metabolism and insulin secretion. Early studies identified an insulin-promoting effect of the LEAP-2 peptide in vitro [51]. The inhibitory effect of LEAP-2 on ghrelin-induced food intake has been observed in lean mice, obese mice, and GHSR-deficient animal models, but the reduction in blood glucose levels was only observed in obese animals. This effect may be due to their hyperglycemic and insulin-resistant state, suggesting that LEAP-2 regulates blood glucose independently of its mechanisms related to food intake control [53]. Bayle et al. investigated the effect of LEAP-2 on insulin secretion using rat pancreatic islet isolation experiments and showed that LEAP-2 regulates insulin by blocking the insulin inhibitory effect of ghrelin. This finding positioned LEAP-2 as a functional antagonist of the pancreatic GHSR1a receptor [61]. A series of experiments further revealed that LEAP-2 regulates insulin secretion and glucose metabolism by binding to the receptor GHSR1a and influencing downstream peroxidase proliferator-activated receptor γ (PPARγ) and glucokinase (Gck) expression levels, providing usable targets for the treatment of type 2 diabetes (T2D) [62,63].

It is worth noting that diabetes is related to the increased level of inflammatory markers, especially in oral diseases such as periodontitis. A bidirectional regulatory relationship between the two was found; hence, it is necessary to maintain oral health through natural substances such as probiotics to control blood glucose regulation [34,64]. Beyond diabetes, polycystic ovary syndrome (PCOS) is a common metabolic and reproductive disease associated with insulin resistance, impaired glucose tolerance, dyslipidemia, and obesity. In women with PCOS, circulating levels of LEAP-2 and ghrelin were found to be decreased, with both hormones showing an independent positive correlation, which contrasted with their previously understood antagonistic effects. These findings indicated that further investigation into LEAP-2 dynamics may provide new insights for treating PCOS [65,66].

## 5. Clinical Status and Medicinal Potential of LEAP-2

### 5.1. LEAP-2 Clinical Application in Obesity and Type 2 Diabetes (T2D)

In the first detailed study examining LEAP-2 regulation in humans, a clear link between LEAP-2 and human metabolic states such as BMI, obesity, and food intake was identified. While these associations are weaker than in mice studies, they nonetheless highlight the significant importance of LEAP-2 in human therapy [67]. It has been shown that only obese patients can detect increased LEAP-2 production, and although this can control behaviors such as overeating, it is not enough to achieve weight loss. Experiments in high-fat mice demonstrated that central injection effectively alleviates negative symptoms compared to systemic administration, suggesting a potential preferred mode of delivery in the future [51,67,68,69]. Hagemann et al. further advanced clinical trials, demonstrating that continuous intravenous injection of LEAP-2 in healthy young men effectively regulates food intake while maintaining stable postprandial blood glucose and lipid levels. The effective concentration of LEAP-2 used in the study was high (at 41.2 ± 1.1 ng/mL), but no safety issues or significant side effects were observed. Clinical trials in overweight individuals have not been conducted, and due to the short half-life of LEAP-2, further research is needed to determine whether adjusting the dose could optimize the therapeutic response [13,70].

### 5.2. LEAP-2 Modulation of Cognition and Memory

The hippocampus is capable of integrating feeding-related signals with cognitive memory processes, in which ghrelin signals can be transmitted to the hindbrain via downstream signals, constituting a hippocampus–hypothalamus–hindbrain pathway that leads to the control of meal size [71]. In the experimental studies of humans and mice at different ages, Tian et al. have found that during ageing, the plasma LEAP-2/acyl-ghrelin molar ratio increased, inhibited hippocampal synaptic function, and impaired neurogenesis, ultimately leading to cognitive deficits. The researchers speculated that reducing the LEAP-2/acyl-ghrelin ratio in older adults to levels seen in younger individuals—potentially through weight gain—might improve memory and delay or even reverse cognitive decline. They further hypothesized that the increasing LEAP-2/acyl-ghrelin molar ratio in the elderly may be an adaptation to systemic metabolic processes during ageing changes. Consequently, LEAP-2/acyl-ghrelin could be used as a biomarker of age-related cognitive decline and balanced as a treatment for age-related cognitive disorders such as Alzheimer’s disease [72].

### 5.3. LEAP-2 Regulation of Growth Hormone Secretion

Adult growth hormone deficiency (aGHD) is caused by congenital or acquired disorders affecting the hypothalamus or pituitary gland, manifesting the reduction in growth hormones, mood disturbances, and various metabolic disorders [73,74]. LEAP-2 levels in adult GHD patients were first investigated by Edoardo Vergani et al. In their trial, plasma LEAP-2 levels in aGHD patients were found to be comparable to those in healthy groups, but the LEAP-2/acyl-ghrelin ratio in aGHD patients was about 1.6 times higher than in healthy controls [75]. More recently, they discovered that ghrelin levels were significantly reduced in aGHD patients, while the LEAP-2/acyl-ghrelin ratio was significantly increased. Studies on neudesin knockout mice revealed physiological effects almost identical to those observed in ghrelin knockout models, such as reduced food intake, increased energy expenditure, and the ability to control both fat and glucose [51,76,77]. Thus, given the antagonistic effect of LEAP-2 with ghrelin, neudesin and LEAP-2 can be considered as functional physiological antagonists, with a significant direct correlation between the two but with a different systemic action than ghrelin [77].

### 5.4. LEAP-2 Regulation of Cardiovascular Function

Obesity induced by a high-fat diet not only leads to insulin resistance but also triggers associated symptoms such as inflammatory responses and myocardial injury through the induction of apoptosis, lipotoxicity, mitochondrial dysfunction, and oxidative stress [78]. Previous studies have demonstrated that ghrelin can inhibit the reduction of obesity-induced myocardial injury by influencing the lncRNA H19/miR-29a/IGF-1 signaling pathway and the HOTAIR/miR-196b/IGF-1 signaling pathway [79,80]. Furthermore, knocking down LEAP-2 has been shown to inhibit M1 macrophage polarization and promote M2 macrophage polarization. This process activates the interaction between GHSR and ghrelin in macrophages, which in turn alleviates obesity-induced myocardial inflammatory injury. Thus, LEAP-2 may represent a new promising therapeutic target for myocardial inflammatory injury [81].

### 5.5. LEAP-2 Treatment of Liver Disease

In patients with severe obesity-associated non-alcoholic fatty liver disease (NAFLD), LEAP-2 has been found to slightly induce the upregulation of factors involved in fibrogenesis such as α-SMA, COL1α1, and TIMP1 via the PI3K/Akt/mTOR signaling pathway. However, co-incubation of LEAP-2 with acyl-ghrelin at specific concentrations can reverse this phenomenon, confirming their antagonistic effects on hepatic stellate cell activation and fibrosis. Similar to vertical sleeve gastrectomy, Roux-en-Y gastric bypass (RYGB) is also a bariatric procedure that not only aids in weight loss but also relieves NAFLD symptoms by altering ghrelin and LEAP-2 concentrations in relation to pro-fibrotic factors. Consequently, the LEAP-2/acyl-ghrelin ratio was expected to be a potential therapeutic target for hepatic fibrosis and liver injury in obese patients [82,83,84]. In recent years, NAFLD has been renamed as metabolic dysfunction-associated fatty liver disease (MAFLD), shifting focus away from the sensitive term “alcohol” and emphasizing metabolic factors that contribute to fatty liver disease, such as obesity, type 2 diabetes, and cardiometabolic abnormalities. Liu et al. identified LEAP-2 as a potent biomarker for differentiating common liver diseases from MAFLD [85].

### 5.6. Role of LEAP2 in Addictive Disorders

Voigt et al. first linked plasma LEAP-2 to behavioral traits, reporting a relationship between LEAP-2 concentration and attentional control. Under fasting conditions, higher LEAP-2 was associated with faster reaction times, suggesting its involvement in impulsive responses and offering new insights into the integration of complex behavioral traits with metabolic signals in humans [86]. Gambling disorder (GD), a condition directly associated with impulsive response, has been identified to involve alterations in the signaling of energy metabolic homeostasis. Specifically, elevated plasma ghrelin and decreased LEAP-2 and adiponectin concentrations have been observed, with statistically lower LEAP-2 concentrations predicting the presence of GD [87]. While the correlation between endocrine factors and clinical features requires further investigation, LEAP-2 presents a promising therapeutic target for GD and other addiction-related disorders, as it can neutralize the potentially harmful effects of ghrelin in impulsive responses [87,88,89]. Recent studies have further clarified the heterogeneity of GD using dual neuropsychological and neuroendocrine profiles, highlighting changes in the concentration of multiple hormones, including LEAP-2, which can help to optimize treatment regimens [90,91].

## 6. Discussion

Since the discovery of LEAP-2, its effective antimicrobial concentration in vitro is significantly higher than the physiological level, demonstrating its efficient antimicrobial capacity [5,29]. Relatively small changes in LEAP-2 levels can substantially affect circulating ghrelin concentrations, allowing LEAP-2 to exert its effects at physiological hormone levels [10]. In this review, we summarized the characteristics of LEAP-2 as both an antimicrobial peptide and an antagonist/inverse agonist of GHSR1a. We provide a systematic comparison of the structure and antimicrobial functions of LEAP-2 to elucidate its mechanisms of action. Furthermore, we analyzed the critical role LEAP-2 plays in energy metabolism by regulating the ghrelin–GHSR1a signaling system, especially in terms of feeding, body weight, and blood glucose. Therefore, it can serve as a potential therapeutic target for fatty liver, diabetes, myocardial injury, and other metabolic diseases [13,70].

However, the clinical application of LEAP-2 is still limited, and further understanding of its mechanism of action and drug modification are needed to reduce potential side effects. Compared to other antibacterial peptides, while LEAP-2 shares similarities in molecular structure, antibacterial mechanisms, and immune regulation, its spectrum of activity and antibacterial efficiency are narrow; therefore, the prospect for human diagnosis and therapy is not clear. For instance, although LEAP-2 in rockfish has good antibacterial activity in vitro, it significantly inhibits the growth of human hepatocytes and has certain cytotoxicity [92,93]. In this case, functional domains and certain amino acids can be replaced and cytotoxicity tests can be performed on different cell lines to obtain the formulation with the best outcome. Further investigation of the LEAP-2 structure at high resolution could facilitate the discovery and design of new functionally selective therapeutic agents to circumvent its structural and functional deficiencies, such as shorter half-life and structural instability. In recent research, the truncated palmitoylated analogue palm-LEAP2 (1–14) shows favorable pharmacokinetics and is expected to have anti-obesity properties. However, it is selectively resistant to obese patients, which means that continuous updating and improvement is still needed to achieve the desired therapeutic purposes [94].

Sodium-glucose cotransporter 2 (SGLT-2) inhibitors are oral drug agents commonly used to treat type 2 diabetes (T2D) and obesity. They help lower blood glucose levels, slightly reduce body weight, and contribute to modest blood pressure reduction. In contrast, glucagon-like peptide 1 (GLP-1) receptor agonists are non-insulin injectable long peptides that regulate insulin and glucagon secretion while controlling appetite. LEAP-2 shares similarities with GLP-1 agonists, but its longevity and potential applications remain unclear. It is hoped that modifications to LEAP-2 or improvements in addressing the clinical risks associated with GLP-1 agonists, such as gastrointestinal side effects, will enable broader clinical use in the future for the benefit of human health [70,95]. Furthermore, in contrast to the more singular roles of metabolic factors like leptin, glucagon, and insulin, LEAP-2 leverages a broad range of functions within a complex regulatory network and mechanism. In addition to the initial clinical application in the treatment of obesity and diabetes, LEAP-2 can also improve cognition and memory, regulate the function of the cardiovascular system, and monitor and even intervene in addictive behaviors, with promising future development prospects [72,77,81,87].

In addition, according to previous studies, LEAP-2 may be the dominant GHSR1a ligand in the feeding state. Since LEAP-2 and ghrelin can bind to the receptor GHSR1a in both competitive and non-competitive manners, their molecular interactions with the receptor need to be carefully considered when developing therapeutic interventions [42,51]. Based on the existing research on the mechanism of LEAP-2, a new receptor (MOSPD2) was recently found in a teleost, *Boleophthalmus pectinirostris*. Experiments showed that the knockdown of MOSPD2 could significantly inhibit the chemotaxis and activation of monocytes/macrophages induced by LEAP-2, as well as its antibacterial activities. Additionally, it can also affect the expression level of several other inflammatory factors [96]. Looking ahead, the identification of additional LEAP-2 receptors is anticipated, with the goal of uncovering signaling pathways independent of the ghrelin–GHSR1a or LEAP-2–MOSPD2 interactions. This could lead to the discovery of new functions for LEAP-2, expanding its potential applications in disease diagnosis and clinical treatment in terms of metabolic regulation.

## Figures and Tables

**Table 1:** LEAP-2 sequences used in this study.

Species	Protein	GenBank Accession No.
*Homo sapiens*	LEAP-2	NP_443203.1
*Sus scrofa*	LEAP-2	NP_998953.1
*Rattus norvegicus*	LEAP-2	NP_001380270.1
*Mus musculus*	LEAP-2	NP_694709.1
*Gallus gallus*	LEAP-2	NP_001001606.1
*Patagioenas fasciata*	LEAP-2	XP_065705459.1
*Chelonia mydas*	LEAP-2	XP_007063679.3
*Podarcis muralis*	LEAP-2	XP_028573793.1
*Rana temporaria*	LEAP-2	XP_040200558.1
*Xenopus tropicalis*	LEAP-2	NP_001106385.1
*Danio rerio*	LEAP-2	NP_001122249.1
*Oryzias melastigma*	LEAP-2	XP_024144419.1
*Scophthalmus maximus*	LEAP-2	XP_035465006.1
*Carassius carassius*	LEAP-2	XP_059379122.1
*Tachysurus fulvidraco*	LEAP-2	XP_026990153.1
*Mobula hypostoma*	LEAP-2	XP_062917438.1
*Petromyzon marinus*	LEAP-2	XP_032813599.1
*Lethenteron camtschaticum*	LEAP-2	OR882686
